# The Influence of Tool Path Strategies on Cutting Force and Surface Texture during Ball End Milling of Low Curvature Convex Surfaces

**DOI:** 10.1155/2014/374526

**Published:** 2014-02-20

**Authors:** Shaghayegh Shajari, Mohammad Hossein Sadeghi, Hamed Hassanpour

**Affiliations:** CAD/CAM and Machining Lab, Manufacturing Group, Faculty of Mechanical Engineering, Tarbiat Modares University, Jalal Ale Ahmad Highway, P.O. Box 14115-111, Tehran, Iran

## Abstract

Advancement in machining technology of curved surfaces for various engineering applications is increasing. Various methodologies and computer tools have been developed by the manufacturers to improve efficiency of freeform surface machining. Selection of the right sets of cutter path strategies and appropriate cutting conditions is extremely important in ensuring high productivity rate, meeting the better quality level, and lower cutting forces. In this paper, cutting force as a new decision criterion for the best selection of tool paths on convex surfaces is presented. Therefore, this work aims at studying and analyzing different finishing strategies to assess their influence on surface texture, cutting forces, and machining time. Design and analysis of experiments are performed by means of Taguchi technique and analysis of variance. In addition, the significant parameters affecting the cutting force in each strategy are introduced. Machining strategies employed include raster, 3D-offset, radial, and spiral. The cutting parameters were feed rate, cutting speed, and step over. The experiments were carried out on low curvature convex surfaces of stainless steel 1.4903. The conclusion is that radial strategy provokes the best surface texture and the lowest cutting forces and spiral strategy signifies the worst surface texture and the highest cutting forces.

## 1. Introduction

CNC milling is today the most effective, productive, and flexible manufacturing method for machining of curved surfaces. Ball end tools are used for machining of 2.5D and 3D surfaces for dies, molds, and various parts, such as aerospace components, due to the fact that the cutter readily adapts well to machining of these parts [1, 2].

The machining of curved surfaces is generally performed in accordance with a given machining strategy. However, as competition grows among manufacturing companies, greater emphasis has been placed on product quality and process efficiency, and this is subsequently promoting and standardizing widespread use of predetermined machining strategy within product design. That is to say, industries require high efficiency machining strategies for curved surface machining, before any machining process to be done, due to the increasing demand for higher accuracy, higher surface integrity, lower machining time, and lower cutting forces. All of these terms resulted from employing an appropriate cutter path strategy.

**Figure 1:**
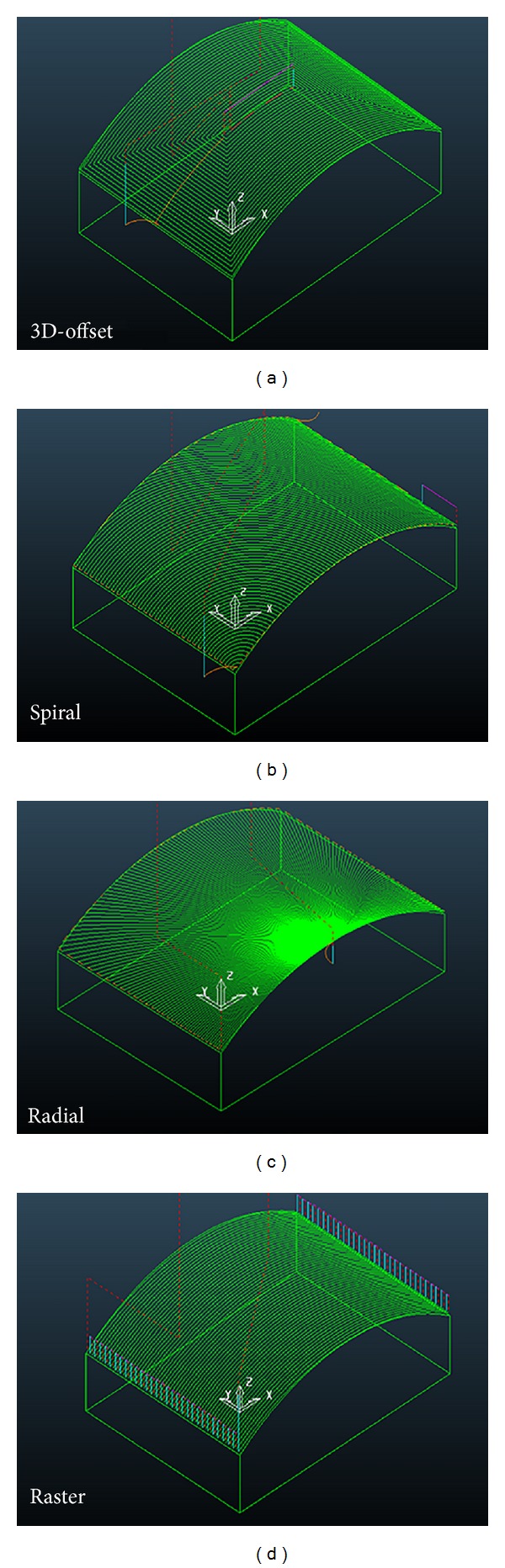
Various 3D-tool path strategies.

Different possible strategies in finish milling can be used. The finish milling operations employed in this study were spiral, radial, 3D-offset, and raster tool paths. Spiral machining creates a spiral tool path from a given focal point while keeping a constant contact between the cutter and workpiece. Radial machining converges tool paths to a central point with the ability to stop short of the center of the radial passes where they become very dense. In raster machining, the passes are parallel in the *XY*-plane and follow the surface in *Z*-direction, in this strategy, in order to reduce machining time, the machining direction offered to be chosen along the long side of the workpiece. In 3D-offset milling, the cutter starts at the periphery to the inner of the surface to be machined or the cutter may start at the center of the workpiece and then proceeds outwards. The cutter recurs to the starting point in each cycle and then cuts outwards to the next outer cycle [3, 4]. It should be noted that in all strategies mentioned above, up and down milling are performed.  Figure 1 illustrates schematically the 3D-tool paths of the strategies tested.

Precision parts with curved surfaces are required in many manufacturing industries. Due to the inherently low stiffness of end mills during manufacturing processes of such parts, cutting forces can cause tool deflections and these deflections have a significant effect on the geometric and dimensional errors in the machined part [5]. Hence, choosing the cutter path strategy in which the lower cutting forces could be resulted might be one method to prevent any catastrophic tool breakage and unfavorable machined surface quality. Ng et al. [6] showed that specific force values when machining with vertical downward strategy are higher than the vertical upward. This explains the extremely short tool life experienced when using this operating mode. Kim et al. [7] conducted simulation and experiments of cutting forces on inclined surfaces and showed that cutting forces were in general lower in horizontal cutter path orientations as compared to milling in vertical cutter path orientations. Chu et al. [8] revealed that although vertical upward orientation at low inclination angles reached at better stability than vertical downward orientation, faster cutting speeds with the former resulted in lower cutting forces. Several researches have also addressed the influence of cutter path on surface roughness, although few studies focus on the impact of tool path strategies on surface texture [9, 10].

Most of the previous researches focused on comparing cutting forces with respect to cutter path orientations, but none of them investigate cutting forces regarding cutter path strategies specially when machining low curvature curved surfaces.

Thus, appraisal of cutter path strategies regarding cutting force, surface texture, and relevant workpiece machining characteristics, when milling of convex surfaces, deserves more merit. Firstly, the objective of this study is to analyze different machining strategies including raster, 3D-offset, spiral, and radial tool paths in 3-axis milling of a low curvature convex geometry. Secondly, the influence of machining parameters on cutting forces based on the tool path strategy employed is investigated and the most significant parameter affecting cutting force in each milling strategy is identified by using analysis of variance (ANOVA). Taguchi design method is also used for the design of experiments. The machining parameters used in this study are cutting speed, feed rate, and step over. Cutting forces and machining time are measured and surface texture is analyzed.

## 2. Experimental Works

The aim of experimental work is to investigate the effect of cutter path strategies and cutting parameters on operating performance when ball nose end milling of a typical low curvature convex surface.

### 2.1. Workpiece Material and Cutting Tool

**Figure 2:**
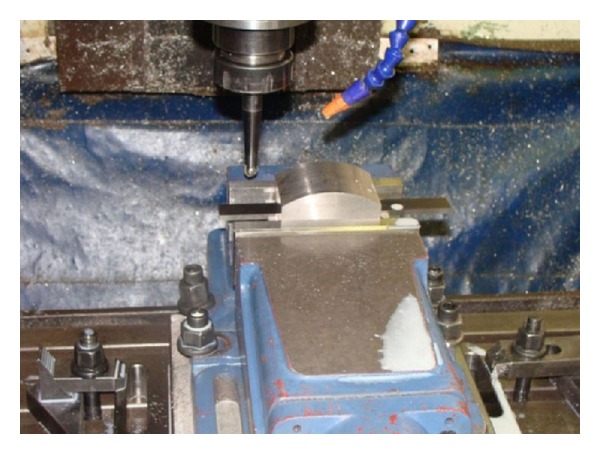
Workpiece specimen's geometry and dimensions.

The workpiece material was X10CrMoVNb9-1DIN stainless steel 1.4903, which is used in turbine and boiler construction, turbine blades, chemical industry, and reactor engineering. Its nominal composition is of 8.26% Cr, 0.91% Mo, 0.37% Mn, 0.29% Si, 0.19 V, 0.15% Cu, 0.13 Ni, 0.11% C, 0.06% Nb, 0.02% W, 0.015% P, and Fe balance (all weights percent).  Table 1 shows the mechanical properties of this material. The workpieces are machined into curved blocks with dimensions of 82 mm × 60 mm × 16 mm (see Figure 2).

**Figure 3:**
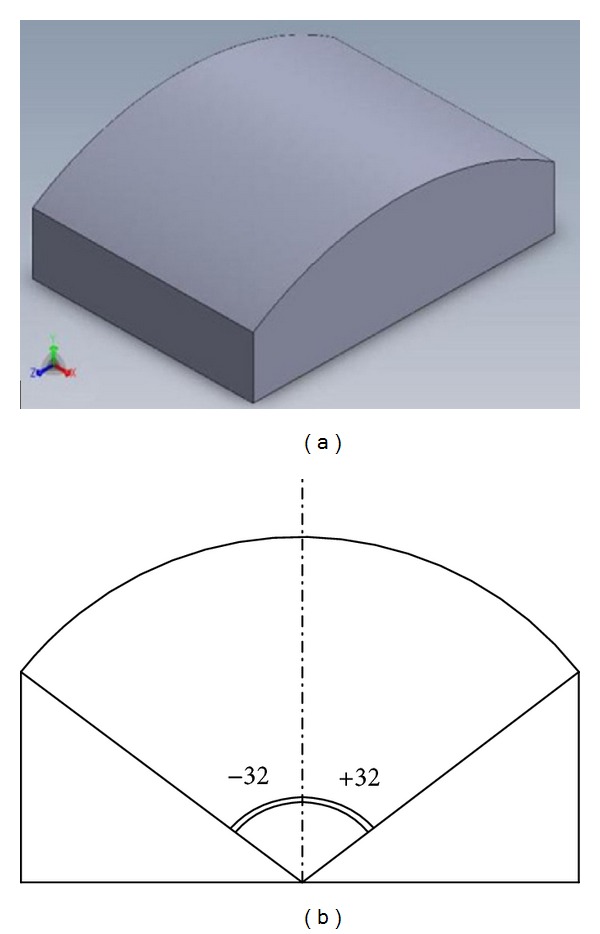
Geometry part used to analyze the milling strategies.

In order to avoid the transient state, the comparison between the strategies took place within a restrictive domain of surface curvature angle (0–32 degrees with respect to *y*-axis). Otherwise, the result of performance characteristics will be changed when using different domains of workpiece surface curvature. The CAD model of the part and the surface curvature angle of parts are illustrated in Figure 3.

The cutting tool selected is two flutes inserted coated carbide ball nose end mill by TiN with 12 mm in diameter made by Walter Company.  Table 2 shows the geometrical properties of the cutting tool. The milling tools are changed after three operations in order to assure that tool wear does not affect the result.

### 2.2. Experimental Equipment and Procedure

**Figure 4:**
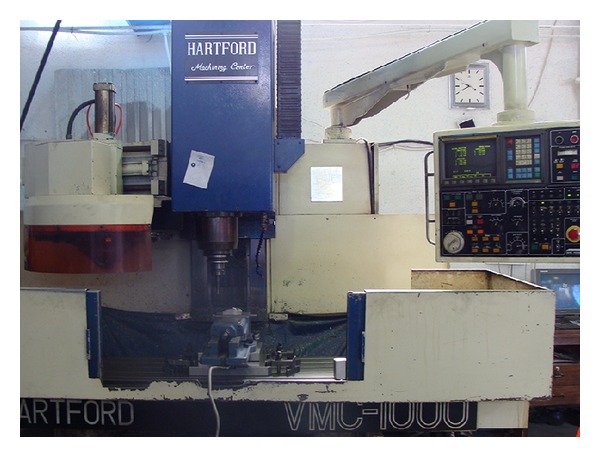
Milling machine.

All machining trials are carried out on a vertical 3-axis CNC machining center HARTFORD with FANUC-OM controller (Figure 4). This has a maximum spindle speed 8000 rpm, maximum power 11 KW, and feed rates up to 10 m/min. A continuous mineral oil based emulsion coolant is employed during machining. Oil is mixed with water to form an emulsion. Emulsion coolant or water soluble coolant is used to reduce friction between the contact metal parts. In addition, whenever removing chips from the workpiece surface was required, air pressure was delivered through a nozzle and directed at the cutting zone.

#### 2.2.1. Cutting Forces and Surface Texture Measurements

Cutting force measurements (*F*_*x*_, *F*_*y*_, and *F*_*z*_) are made using a Kistler 3-component piezoelectric type 9255B platform dynamometer. This has a resonant frequency of 30 KHz in the *x*- and *y*-axes and 30 KHz in the *z*-axis. The dynamometer is connected to a series of charge amplifiers, which in turn are connected to a four-channel oscilloscope with a maximum sampling rate of 3000 M samples/s. The whole system was checked and calibrated prior to use. The cutting force data is downloaded from oscilloscope and information on cutting force signatures is stored onto a PC and after processing of the cutting force data analysis is performed using software.

Surface textures are obtained using an optical microscope Olympus (1000X). The surface roughness is measured by Mahr Roughness Tester at different places in each strategy.

### 2.3. Experimental Design Based on Taguchi Method

The Taguchi method is an experimental design technique, which is useful in decreasing the number of experiments considerably by using orthogonal arrays and also tries to minimize effects of the factors out of control. The basic philosophy of the Taguchi method is to ensure quality in the design phase [11]. The greatest advantages of Taguchi method are to reduce the experimental time, to decline the cost, and to find out significant factors in a shorter period of time. Moreover, Taguchi method employs a special design of orthogonal array to investigate the effects of the entire machining parameters through small number of experiments. Recently, the Taguchi method is widely employed in several industrial fields and research works [12].

Taguchi uses the signal-to-noise (*S*/*N*) ratio as the quality characteristic of choice. Here the term “signal” represents the desirable value (mean) and the “noise” represents the undesirable value (standard deviation). So the *S*/*N* ratio determines the amount of variation that exists in the quality characteristic [13]. There are three types of *S*/*N* ratios according to the objective of quality characteristic (performance characteristic). They include the lower-the-better, the higher-the-better, and the nominal-the better, which are displayed in the following equations.

Smaller the better characteristics:

(1) $$\begin{array}{c} \eta =-10\log\left[\frac{1}{n}\sum_{i=1}^{n}y_{i}^{2}\right]. \end{array}$$

Larger the better characteristics:

(2) $$\begin{array}{c} \eta =-10\log\left[\frac{1}{n}\sum_{i=1}^{n}\frac{1}{y_{i}^{2}}\right]. \end{array}$$

Nominal the better characteristics:

(3) $$\begin{array}{c} \eta =-10\log\left[\frac{1}{n}\sum_{i=1}^{n}\frac{{\overset{-}{y}}_{i}}{S_{y_{i}}^{2}}\right], \end{array}$$

where *η* denotes the *S*/*N* ratio calculated from the observed values (unit: dB), *y*_*i*_ represents the experimentally observed value of the *i*th experiment, *n* is the repeated number of each experiment, and ${\overset{-}{y}}_{i}$ is the average of observed data and *S*_*y*_*i*__^2^ is the variance of *y*. Regardless of the performance characteristic, the larger *S*/*N* ratio corresponds to the better performance characteristic. Therefore, the optimal level of the process parameters is the level with the highest *S*/*N* ratio [14].

The analysis of variance (ANOVA) is used to indicate statistically significant machining parameters and the percent contribution of these parameters on the performance characteristics (output parameters). In fact, ANOVA is a computational technique to estimate quantitatively the relative contribution which each controlled parameter makes on the overall measured response and is expressed as a percentage. Thus information about how significant the effect of each controlled parameter is on the experimental results can be obtained. The total variation in response is the variation due to various controlled factors and due to the error involved in the experimentation. The ANOVA can be done with the raw data or with the *S*/*N* data. Based on the raw data, it signifies the factors which affect the average response rather than reducing the variation. But based on the *S*/*N* data, both of these aspects are taken into account [12]. Therefore, it is used in this research.

### 2.4. Experimental Conditions

The experimental design for each strategy is set according to an L9 orthogonal array based on Taguchi method. The orthogonal array is the L9 (3^3^) that has 9 rows corresponding to the number of experiments (three factors with three levels each). Three factors are determined as controllable cutting parameters including cutting velocity (*V*_*c*_), feed rate (*F*_*c*_), and step over (*S*_*o*_), as shown in Table 3. The levels of each machining condition are determined by taking the cutter and workpiece materials into consideration. The amount of axial depth of cut is set 0.5 mm based on a typical finishing operation and it is fixed throughout all tests. All 9 tests are performed for each strategy and the effect of employing different cutter path strategies when finish milling of stainless steel 1.4903 is investigated.

It is also should be noted that in the field of freeform surface machining, CAM software allows management of various modes of tool path leaning on the geometry of the surface to be machined. Various machining strategies can be used for the same shape. Nevertheless, the choice of a machining strategy remains an expert field [15]. Thus, the cutter path strategies used in this study are simulated on CAM software before machining process.

## 3. Results and Discussion

**Figure 5:**
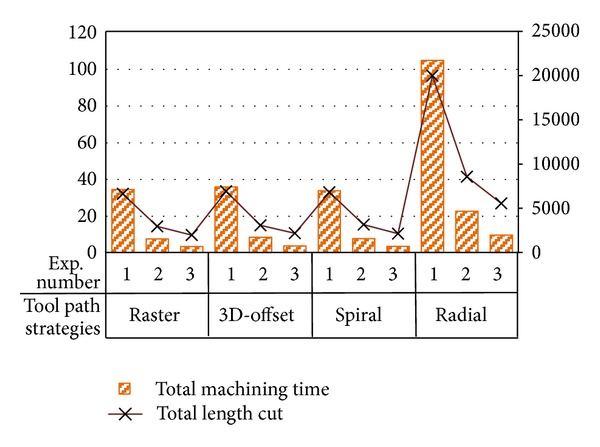
Machining time and length cut versus experiment numbers for different cutter path strategies.

The machining time is measured for each strategy in all experiments (Table 4). Also, the cutting length is attained from CAM software simulation.  Figure 5 provides an exemplary graphical overview of machining time and length cut taking experiments 1 to 3 for each strategy. It can be seen that by increasing feed rate and step over, machining time decreases with length cut regardless of cutter path strategies applied. On the other hand, radial strategy shows higher machining time and length cut in comparison to the other strategies. Furthermore, compared to radial strategy, machining time could be reduced by employing spiral and raster strategies at about 67% and by using 3D-offset strategy at nearly 63%. It should be noted that the machining time difference between 3D-offset, spiral, and raster strategies is partial, which is mainly due to the small dimensions of parts geometry.

**Figure 6:**
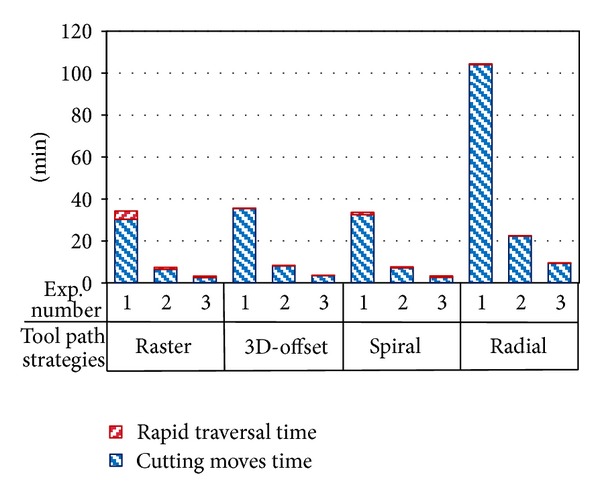
Machining time includes rapid and cutting time separately versus experiment numbers for different tool paths.

In fact, total machining time consists of cutting time and rapid traversal time as illustrated in Figure 6 for different strategies taking the test 1 to 3, for instance. Obviously, rapid traversal time in each strategy includes small portion of total machining time, because cutter path alternations at opposite directions (up and down) result in continuous cutting. Therefore, the machining time difference between different cutter path strategies is not caused by the rapid traversal time variations.

As said elsewhere, the other parameters used in this research to compare the relative advantages of the strategies are the surface texture and the cutting forces.

### 3.1. Cutting Forces

**Figure 7:**
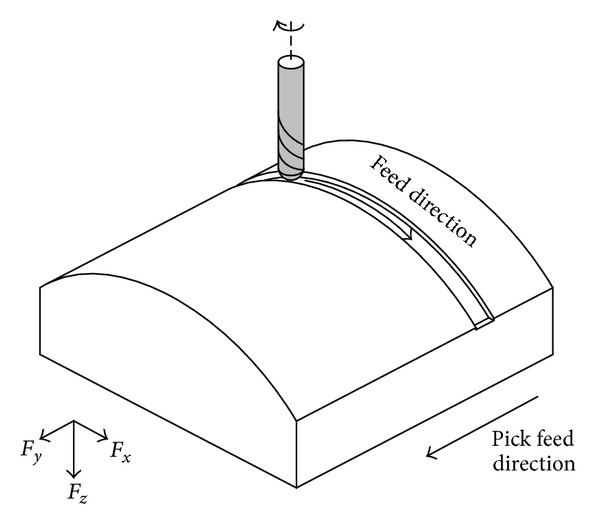
References of cutting force directions.

Cutting forces are the main factors governing machining accuracy, surface quality, machine tool vibration, power requirements, and tool life [5]. Proper selection of the cutter path strategy is crucial in achieving desired machined surfaces. Without considering the impact of appropriate cutter path selection regarding cutting forces, the result can lead to catastrophic cutter failure and therefore lead to unnecessary waste of time, cost, and poor surface quality [16]. In other words, employing cutter path strategies with minimum cutting forces in milling of ruled surfaces can lead to achieve high accuracy and productivity. Therefore, cutting force measurements are carried out to determine the effects of using different tool path strategies in milling of convex surfaces.  Figure 7 depicts the cutting force components, the feed force, pick feed force, and axial force. Three components of cutting force in each strategy fluctuate and consist of many periods. Each period in every strategy determines one track of tool path. Firstly, the maximum absolute value of the cutting force waves in one cutting period is measured. Then, the average values of *F*_max⁡_ amongst all cutting periods in one milling experiment are taken as the three force components *F*_*x*_, *F*_*y*_, and *F*_*z*_.

At last, the resultant cutting force (as *F*_total_) is obtained as determined in (4) for each strategy:

(4) $$\begin{array}{c} F_{\text{total}}=\sqrt{F_{x}^{2}+F_{y}^{2}+F_{z}^{2}}. \end{array}$$

**Figure 8:**
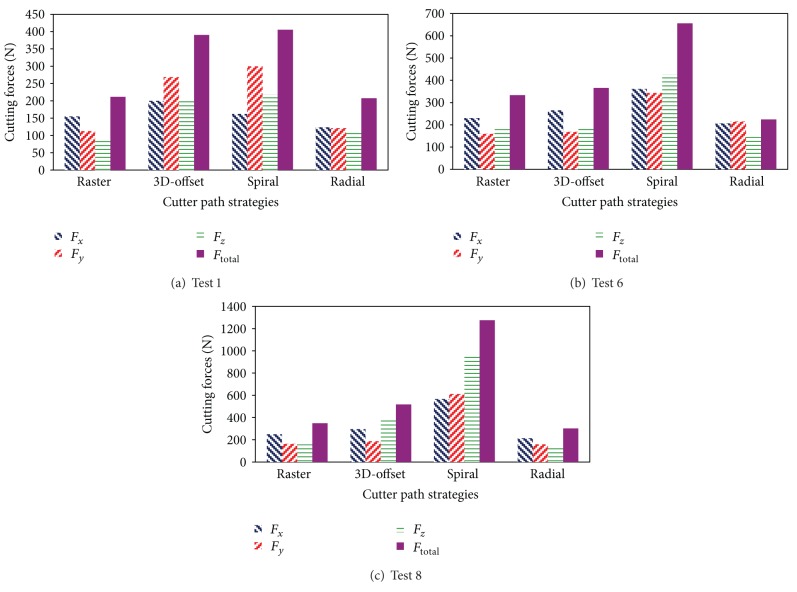
Comparison of cutting force components when employing various milling strategies in (a) test 1, (b) test 6, and (c) test 8.

Table 5 shows the results for total cutting force in all experiments in each strategy. Based on the results, Figure 8 compares the cutting force components obtained when employing various cutter path strategies. As illustrated in this figure, 3 out of 9 experiments having same step over 0.3 mm (tests 1, 6, and 8) are taken, for instance. The comparing criterion is total cutting force. It is observed that when milling with spiral strategy, the highest resultant cutting force is observed followed by 3D-offset, raster, and radial strategies, respectively. Employing radial strategy achieves the lowest cutting force magnitude. Since the comparison between strategies takes place in similar machining conditions, tool-chip contact area is one of the reasons for cutting force differences between different strategies.

In ball nose end milling of inclined surfaces, tool-chip contact area varies significantly when using different cutter path strategies [1]. Cusp height is one of the factors that affect the tool-chip contact area and tool-machined surface contact angle. Since cusp height is the uncut surface region, cusp height regions that remained on machined surface affect the tool-machined surface contact area when cutter moves along the surface. Therefore, the more cusp height is, the more tool-machined surface contact area is expected. According to the previous research, it is shown that mean cusp height in radial machining is the lowest and in spiral machining is the highest amount [17]. Thus, tool-machined surface and tool-chip area is the most for spiral strategy and is the least for radial strategy.

It can also be seen in Figure 8 that cutting force components in 3D-offset strategy are relatively more than raster strategy. It is caused by the abrupt conditions which occur at the cutter entrance and exit conditions at the workpiece. The high impact forces are generated when cutter steps into the workpiece. Because of a sudden increase in chip volume, the in feed executed inside the workpiece results in excessive vibrations due to increased maximum cutting forces.

### 3.2. Surface Textures

**Figure 9:**
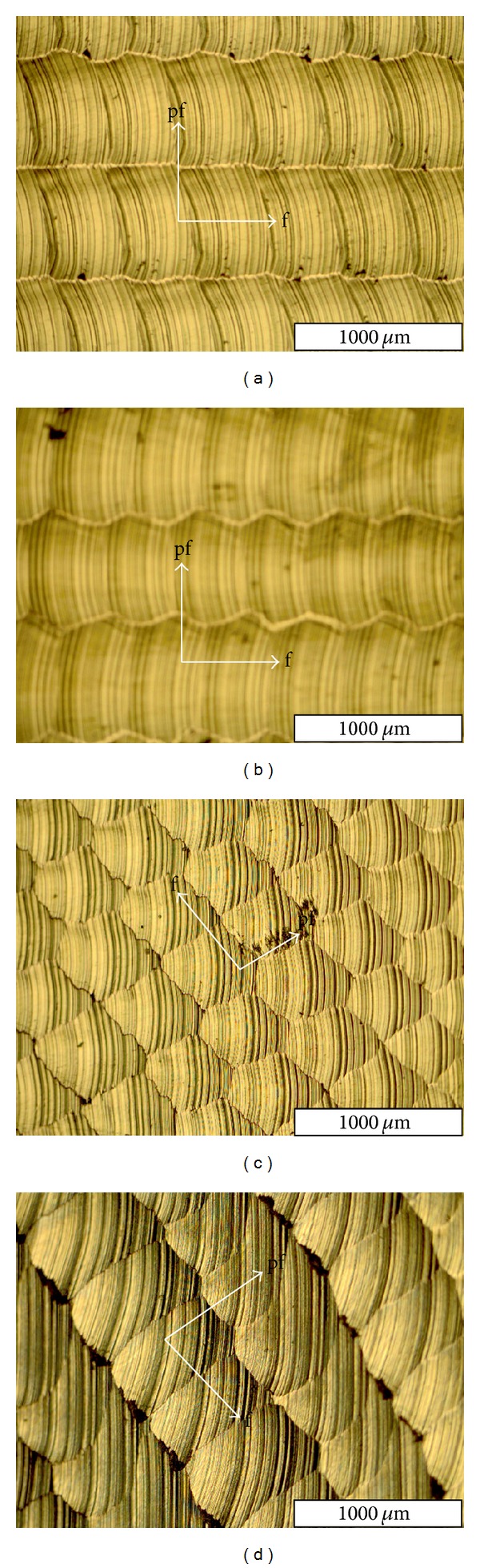
Texture obtained for the (a) raster, (b) 3D-offset, (c) radial, and (d) spiral strategies.

The textures obtained are shown in Figure 9 for raster, 3D-offset, radial, and spiral, respectively (f: feed direction; pf: pick feed direction). The result of texture comparison between the strategies was the same for all the tests (1 to 9); therefore, the result of only one test (test 9) is taken into consideration in this part. The comparison between textures evidences relevant differences. These figures show that there is a markedly texture pattern of crests and gouges provoked by the cutting tool. The crests are due to the tool step over and feed, which correspond to the dark parts of the figure. The gouge patterns also correspond to the bright parts of the figures.

For the radial strategy (Figure 9(c)), traces of feed and pick feed appear uniformly on the machined surface. In addition, the cutting direction in this strategy is not clearly evident. However, the spiral strategy provokes nonuniform traces of feed and pick feed of cutter. The crest patterns in spiral strategy are also wider than the ones obtained from the other strategies. The texture characteristics of the 3D-offset and the raster strategies present approximately similar patterns to each other.

Similarly, the results of surface roughness measurements for each strategy in this test represent approximately the same result as surface texture observations. They include radial 1.261 *μ*m, spiral 2.037 *μ*m, raster 1.793 *μ*m, and 3D-offset 1.514 *μ*m. It is obvious that radial strategy shows the least amount for surface roughness and spiral the highest amount.

### 3.3. Analysis and Discussion of Experimental Results

In this section, results of the cutting experiments are studied by using the *S*/*N* and ANOVA analyses. Afterwards, optimal cutting parameters for cutting forces in each strategy are determined and discussed.

#### 3.3.1. Effect of Control Parameters on Cutting Forces

Total cutting force is set as the objective function of the ball end milling experiment, and the three factors of cutting speed, feed rate, and step over are considered the main machining parameters. Analysis of these selected objective characteristics and the values of the output parameter (total cutting force) allow the calculation to identify which controlling factor is significant for the experiment.

According to the previous sections, the observed values of *F*_*t*_ were examined for each strategy. The levels of each machining parameters were set in accordance with L9 orthogonal array. In this work, the objective function (*F*_*t*_) is minimization. Therefore, the optimal observed *F*_*t*_ is the minimum value. The ratio of signal-to-nose (*S*/*N*) is decided as “the lower is better.” Notably, each experiment in the L9 array is conducted three times for each strategy. Afterwards, the *S*/*N* ratios are calculated from the observed values.

The computed *S*/*N* ratios for each level of cutting parameters affecting the resultant cutting force are presented in Tables 6 to 9 for different cutter paths; lower cutting forces are always preferred. It can be seen from Table 6 that for raster strategy the most influential factor on total cutting force is step over which is then followed by feed rate and cutting speed, respectively. However, in radial cutter path feed rate is considered to be the most significant parameter influencing the total cutting force followed by step over and cutting speed (Table 7). In 3D-offset and spiral strategies, the most prominent factor is cutting speed. Particularly, in 3D-offset, the other parameters only slightly contribute to the evaluation of *F*_total_ (Table 8). In fact, in spiral strategy, the difference between maximum and minimum magnitude of levels (delta) of cutting speed and feed rate is close to each other. Here, step over is seen to be the least important factor. (Table 9)

**Figure 10:**
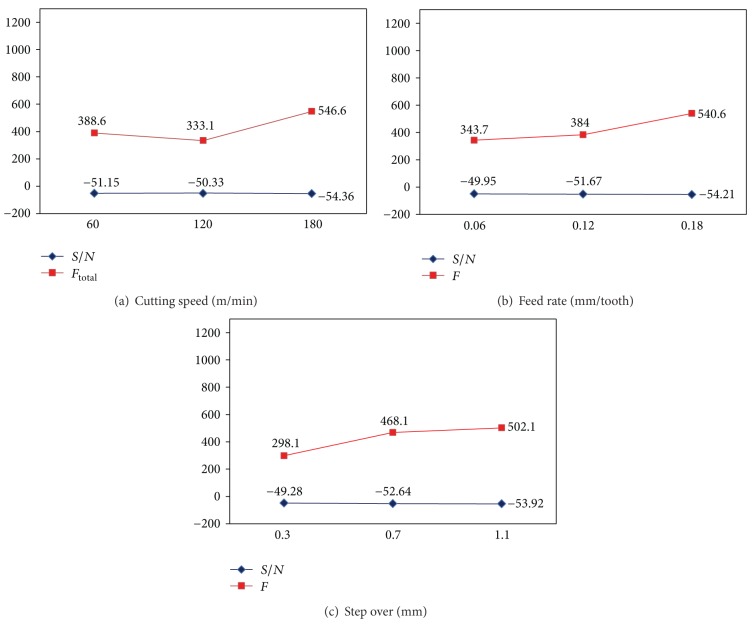
Effects of factors on cutting force for raster cutter path strategy: (a) cutting speed, (b) feed rate, and (c) step over.

**Figure 11:**
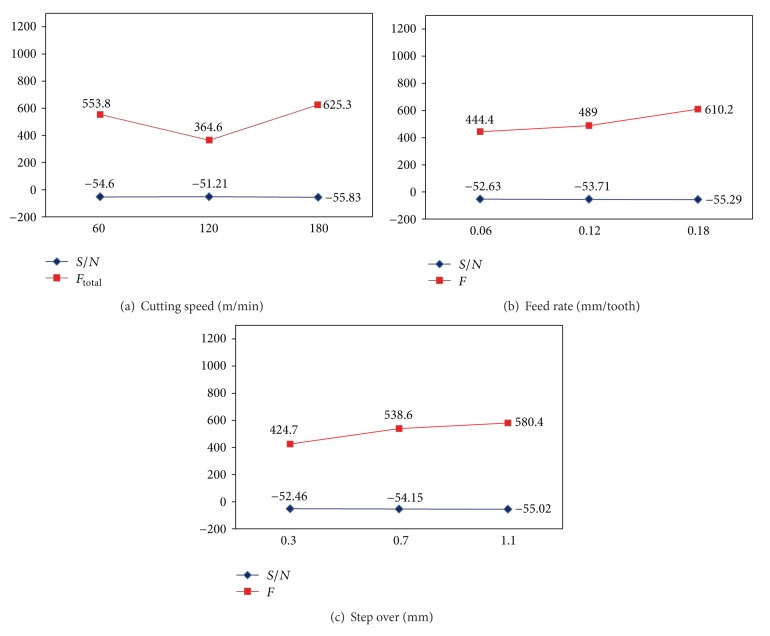
Effects of factors on cutting force for offset cutter path strategy: (a) cutting speed, (b) Feed rate, and (c) step over.

**Figure 12:**
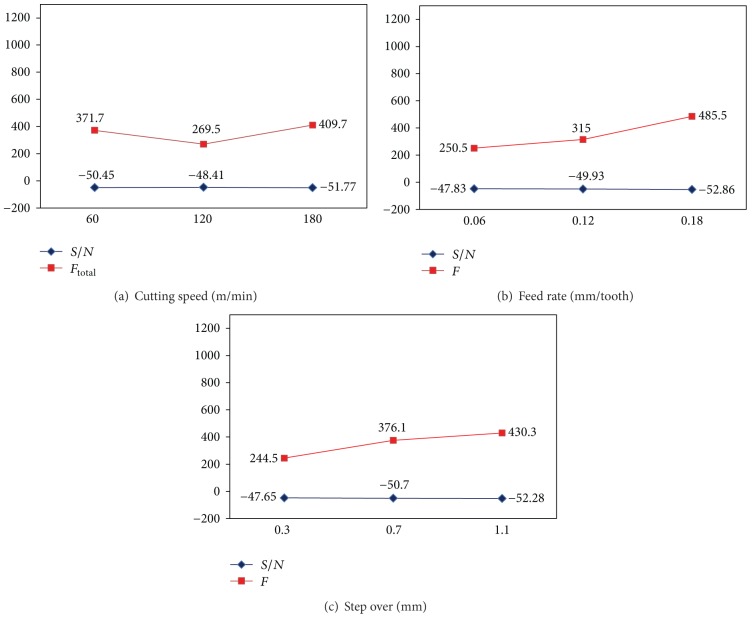
Effects of factors on cutting force for radial cutter path strategy: (a) cutting speed, (b) feed rate, and (c) step over.

**Figure 13:**
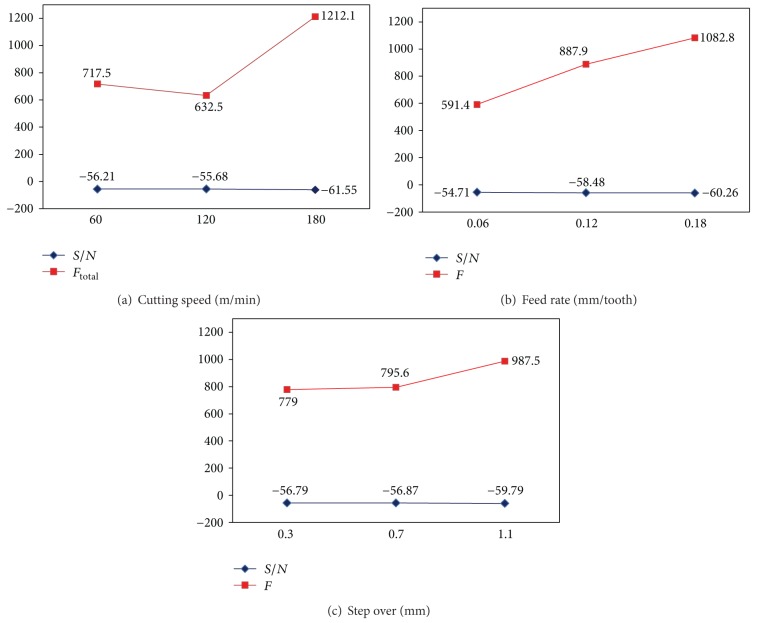
Effects of factors on cutting force for spiral cutter path strategy: (a) cutting speed, (b) feed rate, and (c) step over.

The *S*/*N* ratios are calculated for smaller the better of the resultant cutting forces for each of four cutter paths and then plotted in Figures 10, 11, 12, and 13. From these figures, the effects of each cutting parameter at different level can be observed. According to the analysis, the highest *S*/*N* ratio always yields the optimum quality with minimum variance and reflects the best response given the noise in the machine set-up system, which would be the ideal situation. Therefore, the level with a higher value determines the optimum level of each factor. This is the criteria employed in this study to determine the optimal cutting condition. Therefore, according to the cutting parameters employed, the optimal machining performance for the cutting force is obtained at 120 m/min cutting speed (level 2), 0.06 mm/tooth feed rate (level 1), and 0.3 mm step over (level 1) for all the cutter path strategies. It is emphasized that these conditions only provide the lowest cutting forces among the cutting conditions tested.

Based on the above discussion and also evident from Figures 10–13, the resultant cutting force decrease with minimum step over and minimum feed rate, although increasing cutting speed up to 120 m/min implies smaller cutting force. As speed increases from 60 to 120 m/min, the co-efficient of friction at the chip-tool interface on the rake face decreases which results in an increase in shear plane angle and hence a decrease in shearing area so cutting force decreases. While cutting speed ranges from 120 to 180 m/min, the cutting force increases with increase of cutting speed due to a rise in strain and strain rate; and also work hardening phenomena are occurred. This subsequently leads to a soar in yield strength and resistance of the material and so causes a growth in cutting forces. Moreover, cutting forces increase with increase in feed and step over (radial depth of cut) due to the chip load increase.

#### 3.3.2. Analysis of Cutting Forces

As mentioned earlier, the purpose of the analysis of variance is to establish statistically significant design parameters influencing the output parameter. In fact by using ANOVA, the total variability of the experimental results can be divided into individual components of variance and their significance can be individually assessed. The ANOVA for different factors including total variation, sum of square, sum of mean square, *F* ratio, and *P* value enabled various relative quality effects to be determined.

*Contribution of Each Factor.* The principle of the *F* test is that the larger the *F* value for a particular parameter is, the greater the effect on the performance characteristic is due to the change in that process. Also this analysis is carried out for a level of significance of 5%, that is, for a level of confidence of 95%. Thus, a low *P* value determines that the design factor has a significant effect on whatever is being measured. Here *P* values of less than 5% in ANOVA tables indicate that the process parameter is significant. A value of between 5% and 10% is regarded as mildly significant, while the value of over 10% is regarded as statistically insignificant.

After the end of the tests, results are recorded. The analysis of experimental data for total cutting force and *F* test (standard analysis) is done. This allows identifying the design parameters that significantly affect the overall measured response. Tables 10–13 list the ANOVA and *F* test results associated with *F*_*t*_ obtained from the L9 array for four cutter path strategies.

Table 10 summarizes the correlated results for raster strategy. It indicates the step over as significant factor of influence, with the largest *F* ratio (19.28) and *P* value less than 0.05 (0.49). Here the feed rate and cutting speed are mildly significant with *P* value between 5% and 10%. In radial strategy, since all of *P* calculated values are much larger than 0.05, the significant factors are categorized into two levels: significant (main contribution) and insignificant according to their *F* ratio. Thus, feed rate seems to be more significant with higher *F* ratio (3.79) than other cutting parameters, followed by step over at 3.29; and cutting speed is considered to be insignificant (Table 11).

Table 12 for 3D-offset shows that all of cutting parameters are determined as significant, since the *P* values of all factors are less than 0.05, although the cutting speed is the most significant parameter having the greatest amount of *F* ratio by 643.88 and the least amount of *P* value at 0.002. Similarly, based on Table 13 cutting speed is the most influential input parameter on the cutting force in spiral tool path. This factor accounts for 14.93 of *F* ratio and 0.063 of *P* value. The second factor (feed rate) accounts for 0.081 of *P* value and 11.38 of *F* ratio. The third factor (step over) accounts for 0.194 of *P* value and the least amount for *F* ratio of the experimental variance of cutting force. Thus, it is insignificant.

## 4. Confirmation Tests

The confirmation test is a repetition of the experiment at selected optimal levels of factors [18]. The purpose of the confirmation experiment is to validate the conclusions drawn during the analysis phase. The confirmation experiment is performed by conducting a test with specific combination of the factors and levels previously evaluated [19]. Thus, in this study, once the optimal level of the cutting parameters is identified and the response under these conditions is predicted, the following step is to verify the improvement of the performance characteristics using this optimal combination. One trail for each cutting strategy at the optimal control factor settings is conducted in confirmation experiments. As a result, resultant cutting force is measured for the four cutter path strategies at *V*_*c*2_ *F*_*c*1_ *S*_*o*1_ as optimal cutting conditions.

The results of experiments are presented in Table 14. It shows the results of the confirmation experiment for resultant cutting force values (*F*_*t*exp⁡_), and their *S*/*N* ratios (*η*_exp⁡_) measured using the optimal machining parameters and regarding cutting strategies.  Table 15 represents the difference between the experiment results and predicted values of cutting force (*F*_*t* pred_) and also their *S*/*N* ratios (*η*_pred_) of optimal condition. The predicted *S*/*N* ratios using the optimum levels of machining parameters for resultant cutting force can be calculated from (5) as follows:

(5) $$\begin{array}{c} \eta_{\text{R}\;\text{pred}}={\overset{-}{\eta }}_{\text{R}}+\left({\overset{-}{V}}_{c2}-{\overset{-}{\eta }}_{\text{R}}\right)+\left({\overset{-}{F}}_{c1}-{\overset{-}{\eta }}_{\text{R}}\right)+\left(\overset{-}{S}o_{1}-{\overset{-}{\eta }}_{\text{R}}\right),\\ \eta_{3\text{D}\;\text{pred}}={\overset{-}{\eta }}_{3\text{D}}+\left({\overset{-}{V}}_{c2}-{\overset{-}{\eta }}_{3\text{D}}\right)+\left({\overset{-}{F}}_{c1}-{\overset{-}{\eta }}_{3\text{D}}\right)+\left(\overset{-}{S}o_{1}-{\overset{-}{\eta }}_{3\text{D}}\right),\\ \eta_{\text{RA}\;\text{pred}}={\overset{-}{\eta }}_{\text{RA}}+\left({\overset{-}{V}}_{c2}-{\overset{-}{\eta }}_{\text{RA}}\right)+\left({\overset{-}{F}}_{c1}-{\overset{-}{\eta }}_{\text{RA}}\right)+\left(\overset{-}{S}o_{1}-{\overset{-}{\eta }}_{\text{RA}}\right),\\ \eta_{\text{SP}\;\text{pred}}={\overset{-}{\eta }}_{\text{SP}}+\left({\overset{-}{V}}_{c2}-{\overset{-}{\eta }}_{\text{SP}}\right)+\left({\overset{-}{F}}_{c1}-{\overset{-}{\eta }}_{\text{SP}}\right)+\left(\overset{-}{S}o_{1}-{\overset{-}{\eta }}_{\text{SP}}\right), \end{array}$$

where *η*_R pred_, *η*_3D pred_, *η*_RA pred_, and *η*_SP pred_ are the calculated *S*/*N* ratios at optimal machining conditions and$\;{\overset{-}{\eta }}_{\text{R}}$, ${\overset{-}{\eta }}_{3\text{D}}$, ${\overset{-}{\eta }}_{\text{RA}}$, and ${\overset{-}{\eta }}_{\text{SP}}$ are the average *S*/*N* ratios of all control factors for raster (R), 3D-offset (3D), radial (RA), and spiral (SP) cutting strategies, respectively. ${\overset{-}{V}}_{c2}$ is the average *S*/*N* ratio when the factor *V*_*c*_ (cutting velocity) is at level 2, ${\overset{-}{F}}_{c1}$ is the average *S*/*N* ratio when the factor *F*_*c*_ (feed rate) is at level 1, and $\overset{-}{S}o_{1}$ is the average *S*/*N* ratio when the factor *S*_*o*_ (step over) is at level 1.

From (6), the expression for resultant cutting force values of *F*_*t* R pred_, *F*_*t* 3D pred_, *F*_*t* RA pred_, and *F*_*t* SP pred_ can be derived for raster, 3D-offset, radial, and spiral cutting strategies, respectively:

(6) $$\begin{array}{c} F_{t\;\text{R}\;\text{pred}}=10^{-\eta_{\text{R}\;\text{pred}}/20},\\ F_{t\;3\text{D}\;\text{pred}}=10^{-\eta_{3\text{D}\;\text{pred}}/20},\\ F_{t\;\text{RA}\;\text{pred}}=10^{-\eta_{\text{RA}\;\text{pred}}/20},\\ F_{t\;\text{SP}\;\text{pred}}=10^{-\eta_{\text{SP}\;\text{pred}}/20}. \end{array}$$

From Table 15, which shows comparison of real data with the predicted data, the improvement in cutting force is clearly seen for each cutter path strategy. As this table indicates, improvements in cutting force are provided in raster by 23.1, 3D-offset by 10.59, radial by 33.68, and spiral by 19.11 (N). In addition, the comparison of predicted *S*/*N* ratios of *F*_*t*_(*η*_pred_) with actual values of those (*η*_exp⁡_) shows that the *S*/*N* ratios correlated with the experiment results of *F*_*t*_ are relatively larger than those predicted for the optimal combination levels of machining parameters (see Tables 14 and 15); it is about 1.113 for raster, 0.350 for 3D-offset, 2.223 for radial, and 0.451 (dB) for spiral. Therefore, the experimental results confirm the validity of the used Taguchi method for enhancing the machining performance and optimizing the machining parameters.

## 5. Conclusions

The effect of employing different cutter path strategies on cutting force, surface texture, and machining time when ball end milling of low curvature convex surface of stainless steel 1.4903 was investigated. Moreover, the effect of cutting speed, feed rate, and step over on output characteristics in each strategy was evaluated in accordance with Taguchi method. Some conclusions can be drawn from the study presented.

1. As the data presented in this paper have shown that the use of different cutter path strategies when finishing ball end milling of low curvature convex surfaces has significant effects on the cutting forces, surface texture, and machining time. In general, radial cutter path appears to provide preferred results in terms of more uniform texture and lower cutting forces. However, taking machining time into consideration, the benefits are less clear-cut because of providing the highest total time of machining in comparison to the other tool path strategies. Therefore, when regarding the machining time and cutting force, the general consensus is that raster strategy could be a suitable choice.
2. Resultant cutting force is the highest when using spiral tool path as a consequence of tool-chip contact area. As a result, poor surface texture is obtained regardless of the cutting condition. Overall, the use of spiral cutter path strategy on finish milling of low curvature convex surfaces is not advisable at all.
3. The SNR of resultant cutting forces indicates that the cutting speed (*C*) encompasses the highest effect on the cutting force in 3D-offset and spiral strategies. In contrast, the most influential factor on outer response is step over, followed by feed rate and cutting speed, in that order. However, when employing radial tool path, feed rate seems to be more important, followed by step over.
4. Under determined operating conditions, the minimal cutting forces can be achieved under the following combination of cutting parameters: *V*_*c*_ = 120 m/min, *F*_*c*_ = 0.06 mm/tooth, and *S*_*o*_ = 0.3 mm, regardless of the cutter path strategies employed.
5. The confirmation experiments were conducted to verify the optimal cutting parameters in each strategy. The improvement of cutting force from the predicted results was observed for all cutter path strategies. The lower cutting force achieved from the confirmation runs under the optimal cutting parameters, using those parameter settings with a relatively small number of experimental runs and specific number of control and noise factors, indicates that Taguchi parameter design is an efficient and effective method for optimizing cutting force in a milling operation regardless of cutter path strategies employed.

## Figures and Tables

**Table 1:** Mechanical properties of stainless steel 1.4903.

Thermal conductivity W/m·K	Tensile strength MPa	Hardness HB	Density g/cm^3^	Max temperature in work °C
29.2	630–730	270	7.7	650

**Table 2:** Geometrical properties of cutting tool.

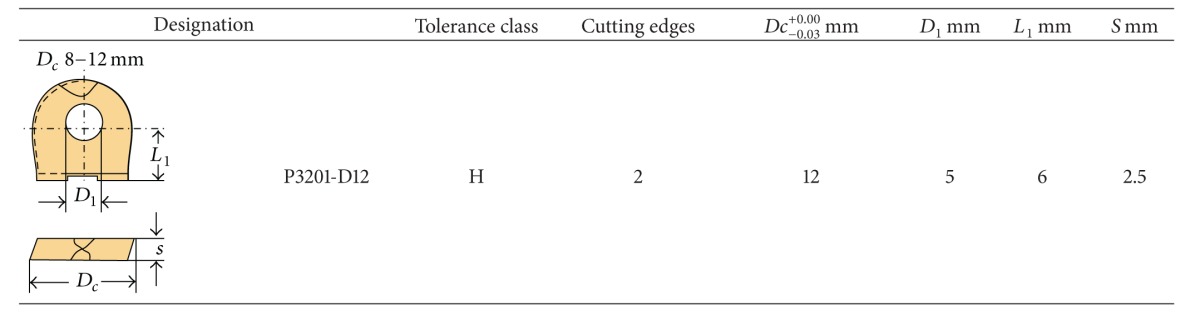

**Table 3:** Machining parameters and their levels.

Control parameters	Symbol		Levels	
		Level 1	Level 2	Level 3
Cutting velocity	*V*_*c*_ m/min	60	120	180
Feed rate	*F*_*c*_ mm/tooth	0.06	0.08	0.12
Step over (radial depth of cut)	*S*_*o*_ mm	0.3	0.7	1.1

**Table 4:** Standard orthogonal array with experiment measurements for machining time.

Sequenced experiment number	Cutting parameters level				Machining time (min)		
	*V*_*c*_ (m/min)	*F*_*c*_ (mm/tooth)	*S*_*o*_ (mm)	Raster	3D-offset	Spiral	Radial
1	60	0.06	0.3	34.09	35.53	33.49	104.29
2	60	0.12	0.7	7.29	8.21	7.45	22.38
3	60	0.18	1.1	3.19	3.52	3.29	9.39
4	120	0.06	0.7	7.29	8.21	7.41	22.39
5	120	0.12	1.1	2.31	2.58	2.42	7.15
6	120	0.18	0.3	5.45	6.20	5.55	17.26
7	180	0.06	1.1	3.19	3.52	3.32	9.44
8	180	0.12	0.3	5.45	6.20	6	17.26
9	180	0.18	0.7	1.43	2.02	1.50	5.03

**Table 5:** Standard orthogonal arrays with experiment measurements for total machining force.

Sequenced experiment number	Cutting parameters level				Total machining force (*F*_*t*_) (N)		
	*V*_*c*_ (m/min)	*F*_*c*_ (mm/tooth)	*S*_*o*_ (mm)	Raster	3D-offset	Spiral	Radial
1	60	0.06	0.3	211.77	390.41	405.50	207.42
2	60	0.12	0.7	402.47	547.74	562.70	287.28
3	60	0.18	1.1	551.6	723.18	1184.40	620.45
4	120	0.06	0.7	265.26	326.38	416.00	228.96
5	120	0.12	1.1	400.52	401.48	825.50	355.52
6	120	0.18	0.3	333.63	365.92	656.00	224.00
7	180	0.06	1.1	554.14	616.46	952.60	315.05
8	180	0.12	0.3	348.93	517.86	1275.50	302.19
9	180	0.18	0.7	736.59	741.59	1408.10	612.00

**Table 6:** Response table for total cutting force for raster strategy.

Level	*V*_*c*_	*F*_*c*_	*S*_*o*_
1	−51.15	−49.95	−49.28
2	−50.33	−51.67	−52.64
3	−54.36	−54.21	−53.92
Delta	4.03	4.26	4.64
Rank	3	2	1

**Table 7:** Response table for total cutting force for radial strategy.

Level	*V*_*c*_	*F*_*c*_	*S*_*o*_
1	−50.45	−47.83	−47.65
2	−48.41	−49.93	−50.70
3	−51.77	−52.86	−52.28
Delta	3.36	5.03	4.63
Rank	3	1	2

**Table 8:** Response table for total cutting force for 3D-offset strategy.

Level	*V*_*c*_	*F*_*c*_	*S*_*o*_
1	−54.60	−52.63	−52.46
2	−51.21	−53.71	−54.15
3	−55.83	−55.29	−55.02
Delta	4.62	2.65	2.56
Rank	1	2	3

**Table 9:** Response table for total cutting force for spiral strategy.

Level	*V*_*c*_	*F*_*c*_	*S*_*o*_
1	−56.21	−54.71	−56.79
2	−55.68	−58.48	−56.87
3	−61.55	−60.26	−59.79
Delta	5.87	5.55	3.01
Rank	1	2	3

**Table 10:** ANOVA for *S*/*N* ratios of cutting forces in raster cutter path strategy.

Source of variance	DOF	Adj SS	Adj MS	*F* ratio	*P* Value
*V*_*c*_ (m/min)	2	27.182	13.5911	15.21	0.062
*F*_*c*_ (mm/tooth)	2	27.566	13.7828	15.43	0.061
*S*_*o*_ (mm)	2	34.451	17.2255	19.28	0.049
Residual error	2	1.787	0.8935		

Total	8	90.986			

**Table 11:** ANOVA for *S*/*N* ratios of cutting forces in radial cutter path strategy.

Source of variance	DOF	Adj SS	Adj MS	*F* ratio	*P* Value
*V*_*c*_ (m/min)	2	17.24	8.618	1.71	0.370
*F*_*c*_ (mm/tooth)	2	38.32	19.162	3.79	0.209
*S*_*o*_ (mm)	2	33.24	16.620	3.29	0.233
Residual error	2	10.11	5.053		

Total	8	98.91			

**Table 12:** ANOVA for *S*/*N* ratios of cutting forces in 3D-offset cutter path strategy.

Source of variance	DOF	Adj SS	Adj MS	*F* ratio	*P* value
*V*_*c*_ (m/min)	2	34.3910	17.1955	643.88	0.002
*F*_*c*_ (mm/tooth)	2	10.6667	5.3333	199.70	0.005
*S*_*o*_ (mm)	2	10.1510	5.0755	190.05	0.005
Residual error	2	0.0534	0.0267		

Total	8	55.2620			

**Table 13:** ANOVA for *S*/*N* ratios of cutting forces in spiral cutter path strategy.

Source of variance	DOF	Adj SS	Adj MS	*F* ratio	*P* value
*V*_*c*_ (m/min)	2	63.284	31.642	14.93	0.063
*F*_*c*_ (mm/tooth)	2	48.268	24.134	11.38	0.081
*S*_*o*_ (mm)	2	17.600	8.800	4.15	0.194
Residual error	2	4.240	2.120		

Total	8	133.391			

**Table 14:** Result of confirmatory experiments.

Cutting strategy	Total Cutting force (*F*_*t* exp⁡_, N)	*S*/*N* for *F*_*t*_(*η*_exp⁡_, dB)
Raster (R)	168.96	−44.556
3D-offset (3D)	256.85	−48.194
Radial (RA)	115.46	−41.249
Spiral (SP)	358.85	−51.098

**Table 15:** Difference between confirmatory test results and calculated values.

Cutting strategy	Predicted values		Comparison	
	*F*_*t* pred_, (N)	*S*/*N* for *F*_*t*_ (*η*_pred_, dB)	|*F*_*t*exp⁡_ − *F*_*t* pred_|	|*η*_exp⁡_ − *η*_pred_|
Raster (R)	192.06	−45.669	23.10	1.113
3D-offset (3D)	267.44	−48.544	10.59	0.350
Radial (RA)	149.14	−43.472	33.68	2.223
Spiral (SP)	377.96	−51.549	19.11	0.451

## References

[1] Toh CK (2004). A study of the effects of cutter path strategies and orientations in milling. *Journal of Materials Processing Technology*.

[2] Daymi A, Boujelbene M, Linares JM, Bayraktar E, Ben Amara A (2009). Influence of workpiece inclination angle on the surface roughness in ball end milling of the titanium alloy Ti-6Al-4V. *Journal of Achievements in Materials and Manufacturing Engineering*.

[3] Krimpenis A, Fousekis A, Vosniakos G (2005). Assessment of sculptured surface milling strategies using design of experiments. *International Journal of Advanced Manufacturing Technology*.

[4] Prabhu PV, Gramopadhye AK, Wang HP (1990). A general mathematical model for optimising NC tool path for face milling of flat convex polygonal surfaces. *International Journal of Production Research*.

[5] Ikua BW, Tanaka H, Obata F, Sakamoto S, Kishi T, Ishii T (2002). Prediction of cutting forces and machining error in ball end milling of curved surfaces—II experimental verification. *Precision Engineering*.

[6] Ng E-G, Lee DW, Dewes RC, Aspinwall DK (2000). Experimental evaluation of cutter orientation when ball nose end milling inconel 718. *Journal of Manufacturing Process*.

[7] Kim GM, Cho PJ, Chu CN (2000). Cutting force prediction of sculptured surface ball-end milling using Z-map. *International Journal of Machine Tools and Manufacture*.

[8] Chu CN, Kim SY, Lee JM, Kim BH (1997). Feed-rate optimization of ball end milling considering local shape features. *CIRP Annals-Manufacturing Technology*.

[9] Ramos AM, Relvas C, Simões JA (2003). The influence of finishing milling strategies on texture, roughness and dimensional deviations on the machining of complex surfaces. *Journal of Materials Processing Technology*.

[10] Toh CK (2004). Surface topography analysis in high speed finish milling inclined hardened steel. *Precision Engineering*.

[11] Asiltürk I, Akkuş H (2011). Determining the effect of cutting parameters on surface roughness in hard turning using the Taguchi method. *Measurement*.

[12] Lin Y-C, Chen Y-F, Wang D-A, Lee H-S (2009). Optimization of machining parameters in magnetic force assisted EDM based on Taguchi method. *Journal of Materials Processing Technology*.

[13] Shaji S, Radhakrishnan V (2003). Analysis of process parameters in surface grinding with graphite as lubricant based on the Taguchi method. *Journal of Materials Processing Technology*.

[14] Nalbant M, Gökkaya H, Sur G (2007). Application of Taguchi method in the optimization of cutting parameters for surface roughness in turning. *Materials and Design*.

[15] Quinsat Y, Sabourin L (2007). Optimal selection of machining direction for three-axis milling of sculptured parts. *International Journal of Advanced Manufacturing Technology*.

[16] Toh CK (2006). Cutter path orientations when high-speed finish milling inclined hardened steel. *International Journal of Advanced Manufacturing Technology*.

[17] Shajari S, Sadeghi MH, Hassanpour H, Jabbaripour B (2012). Influence of machining strategies on Surface roughness in ball end milling of inclined surfaces. *Advanced Materials Research*.

[18] Kandananond K (2009). Characterization of FDB sleeve surface roughness using the Taguchi approach. *European Journal of Scientific Research*.

[19] Tosun N, Cogun C, Tosun G (2004). A study on kerf and material removal rate in wire electrical discharge machining based on Taguchi method. *Journal of Materials Processing Technology*.

